# Dual-Modified A- and B-Type Wheat Starch–PCL Composite Films: Antibacterial and HACCP-Oriented Biodegradable Packaging from Kazakhstani Resources

**DOI:** 10.3390/foods14213730

**Published:** 2025-10-30

**Authors:** Gulnazym Ospankulova, Saule Saduakhasova, Svetlana Kamanova, Dana Toimbayeva, Indira Temirova, Zhainagul Kakimova, Yernaz Yermekov, Berdibek Bulashev, Tultabayeva Tamara, Marat Muratkhan

**Affiliations:** 1 Department of Food Technology and Processing Products, Technical Faculty, Saken Seifullin Kazakh Agrotechnical Research University, Zhenis Avenue, 62, Astana 010011, Kazakhstan; saule_aru@list.ru (S.S.); kamanovasveta@mail.ru (S.K.); bio.dana@mail.ru (D.T.); indira_t85@mail.ru (I.T.); berdibek_aruzhan@mail.ru (B.B.); tamara_tch@list.ru (T.T.); 2 Department of Food Production Technology and Biotechnology, The Engineering-Technological Faculty, Shakarim University, Glinka 20A, Semey 071412, Kazakhstan; zhaynagul.kakimova@mail.ru (Z.K.); yernazyermekov@outlook.com (Y.Y.)

**Keywords:** polycaprolactone, acetylated starch, biodegradable films, antimicrobial activity, composting, HACCP, food packaging

## Abstract

Biodegradable packaging based on starch–polycaprolactone (PCL) composites is a promising route to reduce reliance on petroleum-derived plastics. Here, wheat starches with A- and B-type crystallinity—sourced from Kazakhstani varieties—were dual-modified by electron-beam irradiation followed by acetylation and incorporated into PCL (30–50 wt%) via melt extrusion and compression molding. The resulting films were characterized for morphology, mechanical performance, water-vapor permeability (WVP), thermal behavior, antibacterial activity, and biodegradation under soil and composting conditions. Acetylated A-type starch dispersed more uniformly within the PCL matrix, yielding smoother surfaces, higher tensile strength, and moderate WVP. In contrast, B-type starch produced a more porous microstructure with increased WVP and accelerated mass loss during composting (up to ~45% within 10 days at higher starch loadings). Incorporation of starch slightly decreased thermal stability relative to neat PCL, while agar-diffusion assays against Escherichia coli and Staphylococcus aureus showed loading-dependent inhibition zones, with A-type composites generally outperforming B-type at equivalent contents. Taken together, A-type starch–PCL films are better suited for applications requiring mechanical integrity and controlled moisture transfer, whereas B-type systems favor breathable packaging and rapid compostability. These results clarify how starch crystalline type governs structure–property–degradation relationships in PCL composites and support the targeted design of sustainable packaging materials using regionally available starch resources.

## 1. Introduction

Plastic pollution and its environmental consequences have accelerated the search for biodegradable alternatives to petroleum-derived packaging materials. Conventional plastics are persistent in soil and aquatic environments, threatening ecosystems and food safety. To address this challenge, researchers have increasingly focused on biodegradable polymers such as polycaprolactone (PCL) and starch-based composites [1,2,3,4,5].

PCL is a synthetic aliphatic polyester with good film-forming ability, flexibility, and biocompatibility [6,7]. However, its relatively high cost and slow degradation rate under natural conditions restrict large-scale application [8]. Blending PCL with natural polymers has been widely adopted to lower production cost and accelerate biodegradability [9,10]. Starch is particularly attractive due to its abundance, low price, renewability, and favorable film-forming properties [11,12]. Nevertheless, the high hydrophilicity of native starch and its incompatibility with hydrophobic polymers such as PCL often lead to phase separation, weak interfaces, and poor water resistance [13,14].

To overcome these drawbacks, starch modification has been extensively studied. Dual modification strategies that combine physical methods (e.g., electron-beam irradiation) with chemical treatments (e.g., acetylation) improve hydrophobicity, stability, and compatibility with synthetic polymers [14,15]. Most previous studies have concentrated on starches from corn, potato, or cassava [16,17,18], while the role of crystalline type (A vs. B) in determining composite film performance remains underexplored. In particular, wheat starch—an underutilized but abundant crop resource in Kazakhstan—provides a unique opportunity to examine how crystalline structure influences composite behavior.

Based on starch structure–property relationships, we hypothesize that acetylated A-type wheat starch, with its compact crystalline packing, disperses more uniformly in the PCL matrix and improves tensile strength and barrier properties. By contrast, acetylated B-type starch, with looser crystallinity and smaller granule size, is expected to promote higher water vapor permeability and faster biodegradation.

The objective of this study was therefore to develop and characterize starch–PCL composite films containing dual-modified A- and B-type wheat starches. Using melt extrusion and compression molding, films with varying starch contents were produced and systematically evaluated for physicochemical, morphological, thermal, antibacterial, and biodegradation properties. In addition, by linking film functionality with antimicrobial performance, the work provides preliminary insights relevant to HACCP-based food safety management, where packaging can serve as an auxiliary preventive measure at critical control points.

## 2. Materials and Methods

### 2.1. Materials

Dual-modified wheat starches (A-type and B-type) were obtained from the fractionation of Kazakhstani wheat according to Muratkhan et al. (2025) [16]. Native corn starch (Zharkent Starch Factory, Zharkent, Kazakhstan) was used only for preliminary comparison. Polycaprolactone (PCL, Mw 80,000, ≥99% purity, Sigma-Aldrich, Rockville, MD, USA) was selected as the main polymer. Analytical-grade reagents (acetic anhydride, NaOH, HCl, glycerol, chloroform) were used without further purification. Neat PCL films were prepared as controls.

### 2.2. Starch Modification

Starch modification was carried out in two steps:

Physical treatment: Native wheat starch was irradiated with electron beams (9 kGy, 5 MeV, 1 mA) at the National Nuclear Center of Kazakhstan (ILU-10 accelerator, Budker Institute, Novosibirsk, Russia).

Chemical modification: Acetylation was performed following Kumoro et al. [18] with minor adaptations [19]. Briefly, 100 g of irradiated starch were suspended in distilled water (1:5, *w*/*v*), adjusted to pH 8.0, and reacted with acetic anhydride (1:5 *w*/*w*, 50 °C, 40–60 min). The product was neutralized, washed, dried, milled, and sieved (≤250 μm).

### 2.3. Determination of Acetyl Group Content and Degree of Substitution

*Acetyl* content and the degree of substitution (*DS*) were determined titrimetrically according to Würzburg (1986) [20]. One gram of acetylated starch was suspended in 75% ethanol, heated, treated with NaOH, and back-titrated with 0.5 N HCl. Calculations followed standard equations:

(1) $$\mathrm{Acetyl}\;\left(\%\right)=\frac{\left(V\;blank-V\;sample\right)\times N\times 0.043\times 100}{m}$$

(2) $$DS=\frac{162\times \mathrm{Acetyl}\;(\%)}{4300-(42\times \mathrm{Acetyl}\;(\%))}$$

where *V* is the titrant volume (mL), *N* is the normality of HCl, and m is the sample mass (g).

### 2.4. Determination of Solubility and Swelling Capacity

Solubility and swelling power were measured by dispersing 0.5 g of starch in 20 mL distilled water, heating at 85 °C for 30 min, and centrifuging at 3000 rpm for 15 min. Solubility was expressed as the dried supernatant mass, while swelling power was calculated as sediment weight relative to dry starch [21].

### 2.5. Determination of Physicochemical Properties

Physicochemical parameters were determined according to national and international standards. Moisture content was measured by oven-drying at 105 °C according to GOST 26312.7-84 and ISO 712:2009 [22,23]. Ash content was obtained by incineration at 600 °C following GOST 26312.10-84 and ISO 3593:1981 [24,25]. The pH value was measured in 10% starch suspensions after 30 min of equilibration according to GOST 26312.15-84 [26]. Bulk density was calculated as the mass-to-volume ratio in a 100 mL cylinder based on GOST R 55865-2013 [27]. Water and oil absorption capacities (WAC and OAC) were determined following GOST R 55486-2013 [28].

### 2.6. Scanning Electron Microscopy (SEM)

Film specimens (3 × 3 mm) were cut from the prepared starch-based films, cryo-fractured in liquid nitrogen to obtain clean cross-sections, and mounted on aluminum stubs using double-sided carbon tape. Samples were sputter-coated with a thin layer of gold (~10 nm) using a sputter coater (SC7620, Quorum Technologies Ltd., Lewes, UK) to ensure conductivity. Morphological observations were carried out using a scanning electron microscope (JCM-7000, JEOL, Tokyo, Japan) operated in high-vacuum mode at an accelerating voltage of 15 kV. Micrographs were acquired at magnifications of 800× and 3000× for cross-sectional analysis, while film surfaces were additionally examined at 500× and 2000× to evaluate surface morphology.

### 2.7. Film Preparation and Optimization

*Casting trials (preliminary stage).* Modified starch dispersions (3 g/100 mL) were prepared by gelatinization at 85 ± 2 °C for 20 min, followed by cooling to 65 °C. Glycerol or poly (vinyl alcohol) (PVA) was subsequently incorporated at concentrations corresponding to 10–40% (*w*/*w*, based on starch). The resulting dispersions were poured into Petri dishes and dried at 50 °C for 24 h. This preliminary casting stage was performed to evaluate the effects of plasticizer type and concentration on film flexibility and mechanical resistance. The preliminary results indicated that 10% glycerol achieved the most favorable balance between flexibility and tensile strength. This concentration was therefore selected for the subsequent extrusion process.

*Extrusion–molding (main stage).* Based on the optimal plasticizer concentration identified above, acetylated A- and B-type starches were blended with poly(ε-caprolactone) (PCL) and glycerol in a twin-screw extruder operated at 90–150 °C and 50 rpm. The extrudates were pelletized and subsequently hot-pressed at 150 °C under 5 MPa for 5 min, followed by cooling under pressure. Neat PCL films were prepared under identical conditions and used as controls. Extrusion–molding was employed as the primary processing method to enhance interfacial compatibility between starch and PCL and to obtain formulations suitable for potential industrial applications [29,30].

### 2.8. Evaluation of Film Properties

The properties of the composite films were evaluated using standardized methods. Mechanical properties, including tensile strength and elongation at break, were determined with a universal testing machine (Instron 3369, Instron Corp., Norwood, MA, USA) in accordance with ASTM D882 [31], while film thickness was measured at ten random positions using a digital micrometer. Barrier performance was assessed by water vapor permeability (WVP) using the gravimetric method according to ASTM E96-00 [32]. Optical transparency was measured at 600 nm with a UV–Vis spectrophotometer. Thermal behavior was analyzed by differential scanning calorimetry (DSC, Q2000, TA Instruments, New Castle, DE, USA), conducted from 20 to 200 °C under a nitrogen atmosphere at a heating rate of 10 °C/min, and thermogravimetric analysis (TGA, Q50, TA Instruments), performed from 25 to 600 °C at 10 °C/min with a nitrogen flow rate of 50 mL/min. These protocols followed established procedures for starch–polymer composite films [29,30,31].

### 2.9. Antibacterial Activity Test

The antibacterial activity of the composite films was evaluated against Escherichia coli (ATCC 25922) and Staphylococcus aureus (ATCC 6538) using the agar diffusion method according to ISO 22196:2011 with minor modifications [33]. Briefly, circular film samples (20 mm diameter) were sterilized under UV light for 30 min and placed on nutrient agar plates previously inoculated with 0.1 mL of bacterial suspension (approximately 10^6^ CFU/mL). The plates were incubated at 37 °C for 24 h, after which the diameters of the inhibition zones (including the film disk) were measured in millimeters using a digital caliper. Each test was performed in triplicate, and the results were expressed as mean ± standard deviation. Sterile PCL films served as the negative control.

### 2.10. Evaluation of Biodegradability (Soil Burial and Controlled Composting)

Biodegradation was assessed under (i) natural soil burial and (ii) controlled composting, following ISO 17556:2019 and ASTM D5338-15 [34,35]. Natural soil (sandy loam; pH 6.8; organic matter 2.4%) was collected from an agricultural field, sieved to 2 mm, and maintained at 25 ± 2 °C and 60% water-holding capacity. Composting tests were conducted in an incubator (INE 600, Memmert, Schwabach, Germany) at 58 ± 2 °C and 55–60% relative humidity. Film specimens (~20 × 20 mm) were dried to constant mass (*W*_0_), buried at ~20 mm depth, retrieved at predefined intervals (soil: days 0, 10, 20, 30; compost: days 0, 5, 10, 30), gently cleaned, dried, and weighed (*Wt*). Mass loss (%) was calculated as:

(3) $$\mathrm{Mass}\;\mathrm{lose}\;(\%)=\frac{\;W_{0}-Wt}{W_{0}}\times 100$$

where *W*_0_ is the initial dry weigh and *Wt* is the mass at time *t*. Weight loss data were collected periodically (every 10 days for soil, every 5 days for compost), and films were examined under a stereomicroscope (MZ95, Leica, Wetzlar, Germany) for morphological changes.

### 2.11. Statistical Analysis

All experiments were conducted in triplicate, and results are expressed as mean ± standard deviation. One-way ANOVA was applied to detect significant differences between treatments. Duncan’s multiple range test (*p* < 0.05) was used for all-sample comparisons, while Dunnett’s test was applied when comparing to the PCL control. Statistical analyses were performed using SPSS Statistics 19.0 (SPSS Inc., Chicago, IL, USA).

## 3. Results

### 3.1. Physicochemical Properties of Native and Modified Starches

#### 3.1.1. Physicochemical Properties of Native and Modified Starches

The physicochemical parameters of native and dual-modified starches are summarized in Table 1. Moisture content ranged from 9.5–11.4%, which is consistent with reported values for cereal starches [28,29,32,33]. Ash content (0.12–0.24%) also remained within food-grade standards, indicating that dual modification did not introduce mineral impurities. A slight reduction in bulk density was observed after irradiation–acetylation, suggesting partial disruption of granule packing and loosening of starch structure, as also reported for irradiated potato and cassava starches [36,37].

The pH of native starch suspensions (10%, *w*/*v*) was close to neutral (6.54–6.72), and decreased slightly following acetylation (6.41–6.55), likely due to the introduction of acetyl groups. Water and oil absorption capacities (WAC and OAC) increased significantly (*p* < 0.05) after irradiation–acetylation, indicating improved hydrophilicity and enhanced interaction with hydrophobic solvents, respectively. These changes confirm the effectiveness of dual modification in altering functional behavior at the molecular level [38,39].

#### 3.1.2. Solubility, Swelling Power, and Degree of Substitution

The functional properties of dual-modified starches are presented in Table 2. Irradiation enhanced solubility and swelling power by partially disrupting crystalline regions, thereby facilitating acetyl group substitution. Among the samples, A-type wheat starch exhibited the highest degree of substitution (DS = 1.95), whereas B-type wheat starch showed lower substitution efficiency (DS = 1.08). This is consistent with its more compact crystalline arrangement, which restricts esterification sites [7,39]. Corn starch showed only moderate DS values (0.88), confirming wheat starch as a more reactive substrate.

These findings agree with the reports of Cheng et al. [7], who demonstrated that crystalline type strongly influences chemical reactivity during starch esterification. The higher substitution of A-type starch enhances its functional diversity, while B-type starch contributes higher swelling capacity (7.76%) due to its looser crystalline lattice and greater water penetration, consistent with Ulfa et al. [21]. This complementary functionality highlights the potential for tailoring A- and B-type starches toward specific biodegradable packaging applications, with A-type suited for improved stability and B-type for enhanced degradability.

### 3.2. Plasticization and Mechanical Properties of Acetylated Starch Films (Casting Stage)

To improve flexibility, acetylated starch films were plasticized with glycerol or polyvinyl alcohol (PVA) at concentrations of 10–40% (*w*/*w* relative to starch). As shown in Table 3, elongation at break decreased with increasing plasticizer concentration for all starch types, reflecting the well-known plasticization–strength trade-off in starch films [37,40].

Films plasticized with glycerol exhibited significantly higher elongation compared to PVA, consistent with glycerol’s smaller molecular size and stronger hydrogen bonding with starch chains, which increases chain mobility [36]. This trend aligns with previous observations in cassava and sugar palm starch films, where glycerol effectively enhanced flexibility but excessive addition promoted brittleness [18,38].

At 10% glycerol, B-type wheat starch films achieved the highest elongation (48.32%), which can be explained by their looser crystalline packing that allows more efficient plasticizer penetration. However, at 30–40% glycerol, all films became brittle due to excessive plasticizer disrupting the polymer network, consistent with findings from maize starch films [18,38].

Therefore, 10% glycerol was identified as the optimal plasticizer level for subsequent extrusion, balancing mechanical strength with flexibility. From a practical perspective, this level ensures elasticity suitable for handling and packaging applications, while avoiding the structural weakness associated with higher plasticizer concentrations [40,41,42,43,44,45,46].

### 3.3. Composite Formulations and Mechanical Properties of PCL–Starch Films (Extrusion Stage)

The incorporation of dual-modified starch into PCL composites significantly influenced mechanical strength. Regression analysis for B-type starch composites (Table 4) showed that starch content had a negative impact on tensile strength, while glycerol and CaCO_3_ exerted positive contributions. This trend is consistent with previous reports that increasing starch loading weakens the polymer matrix by introducing rigid domains and poor interfacial adhesion [47]. Conversely, glycerol enhanced flexibility by improving polymer chain mobility, while CaCO_3_ functioned as a reinforcing filler, improving stress transfer within the matrix, in line with findings from Ortega-Toro et al. [47].

The optimal formulation (30–40% starch, 10% glycerol, 5% CaCO_3_) yielded tensile strengths between 21–28 MPa, values comparable to conventional synthetic films [46]. Validation confirmed the robustness of the regression model (R^2^ > 0.97), further supporting the practical utility of the design-of-experiments approach for predicting composite performance.

Interestingly, A-type starch composites exhibited a stronger reinforcement effect from CaCO_3_ (β = +8.654) compared to B-type starch, which can be explained by the denser crystalline packing of A-type starch that improved filler dispersion. This suggests that A-type starch composites may provide better mechanical stability for medium-term packaging, while B-type starch composites, though mechanically weaker, may be more suitable for short-term or compostable packaging due to their higher degradability.

### 3.4. Optical Transparency of Composite Films

Optical transparency decreased with increasing starch concentration, confirming that starch incorporation compromises film clarity (Table 5). At 600 nm, neat PCL films exhibited the highest transparency (91.3%), while starch composites ranged from 74.6% (A30%) to as low as 41.5% (B50%). The reduction is attributed to light scattering from starch granules and interfacial heterogeneities within the PCL matrix, consistent with SEM results showing non-uniform dispersion. Similar decreases in transparency with starch addition have been reported for PCL–starch and starch–PLA composites [36,38].

At equivalent starch levels, A-type starch composites maintained higher transparency than B-type, which may be due to their denser crystalline structure and smaller granule swelling, leading to more homogeneous dispersion in the polymer matrix. Glycerol addition slightly improved transparency, likely by reducing microvoids and enhancing polymer–starch compatibility, a trend also reported in sugar palm and maize starch films [36,38].

From an application standpoint, transparency values above ~60% (≤40% starch loading) are still acceptable for many food packaging uses, particularly for products where product visibility is important (e.g., fresh produce). However, higher starch loadings (>50%) may limit their application to non-visual or compostable packaging where clarity is not a primary requirement.

### 3.5. Morphological Characterization of Composite Films

**Figure 1:**
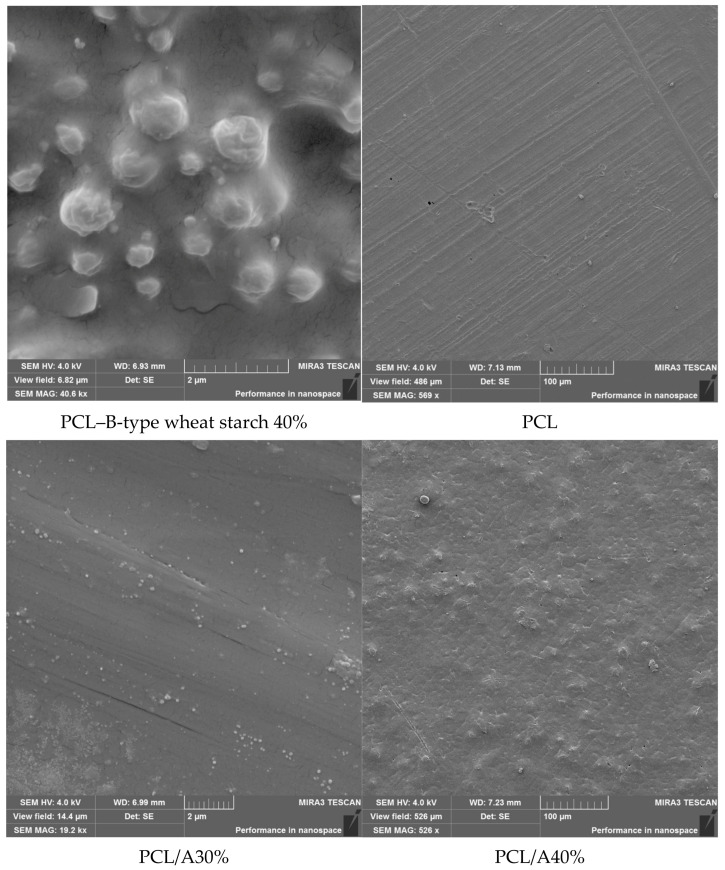

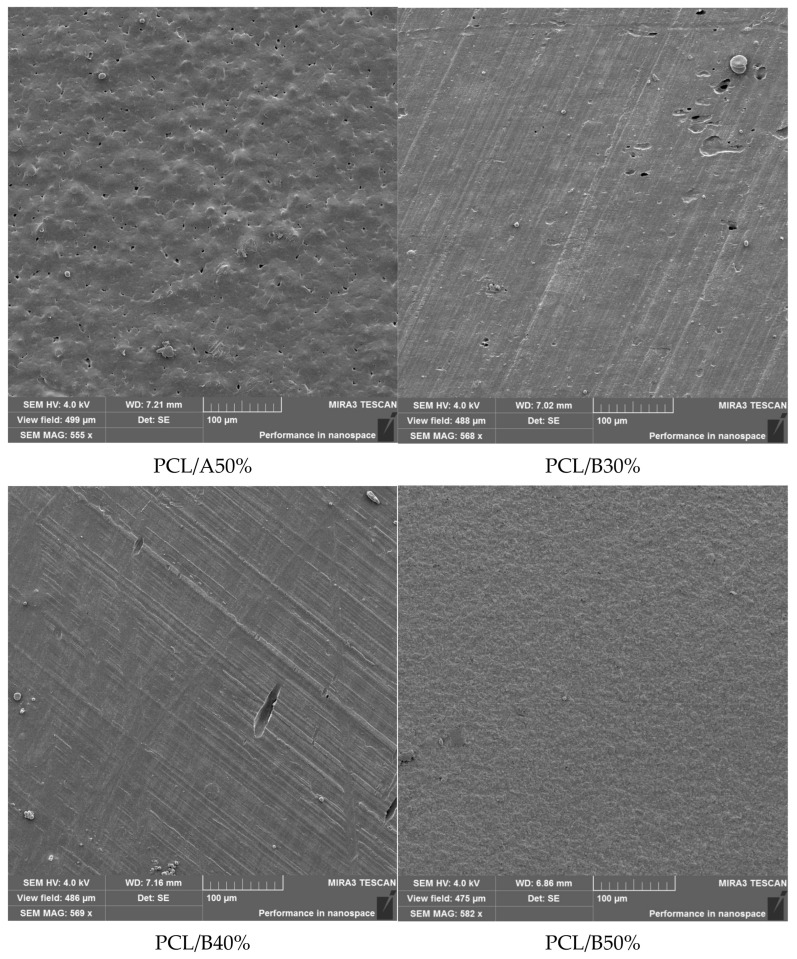
SEM micrographs of starch–PCL composite films with varying starch types and concentrations.

SEM analysis (Figure 1) revealed distinct morphological differences between neat PCL and starch–PCL composites. Pure PCL films exhibited smooth, compact, and homogeneous surfaces, consistent with its semicrystalline structure and high polymer compatibility. Similar morphologies were reported in neat PCL films by Ortega-Toro et al. [48] and Avella et al. [40].

Incorporation of dual-modified starch introduced surface roughness, voids, and interfacial discontinuities, which became more evident at higher starch loadings (40–50%). These morphological changes indicate partial phase separation and weaker interfacial adhesion between starch and PCL domains, consistent with earlier reports on starch-filled polyester composites [45,46].

Interestingly, A-type starch composites (PCL/A30–50%) exhibited relatively uniform dispersion and compact structures compared with B-type starch composites. This suggests better compatibility of A-type starch with PCL, possibly due to its denser crystalline packing and higher degree of substitution achieved during acetylation, which improved polymer–starch miscibility [49]. Conversely, B-type starch films displayed more porosity, microcracks, and heterogeneity, consistent with their higher swelling power and smaller granule size, which facilitated channel formation within the matrix. Such features, while compromising mechanical strength, are expected to enhance water vapor permeability and biodegradation [46].

These morphological observations support earlier mechanical and permeability data: A-type starch enhances film stability, while B-type starch promotes porosity and faster degradation. This indicates that starch crystalline type can be exploited to tailor packaging films for durability (A-type) or rapid compostability (B-type).

### 3.6. Thermal Degradation Profiles

**Figure 2:**
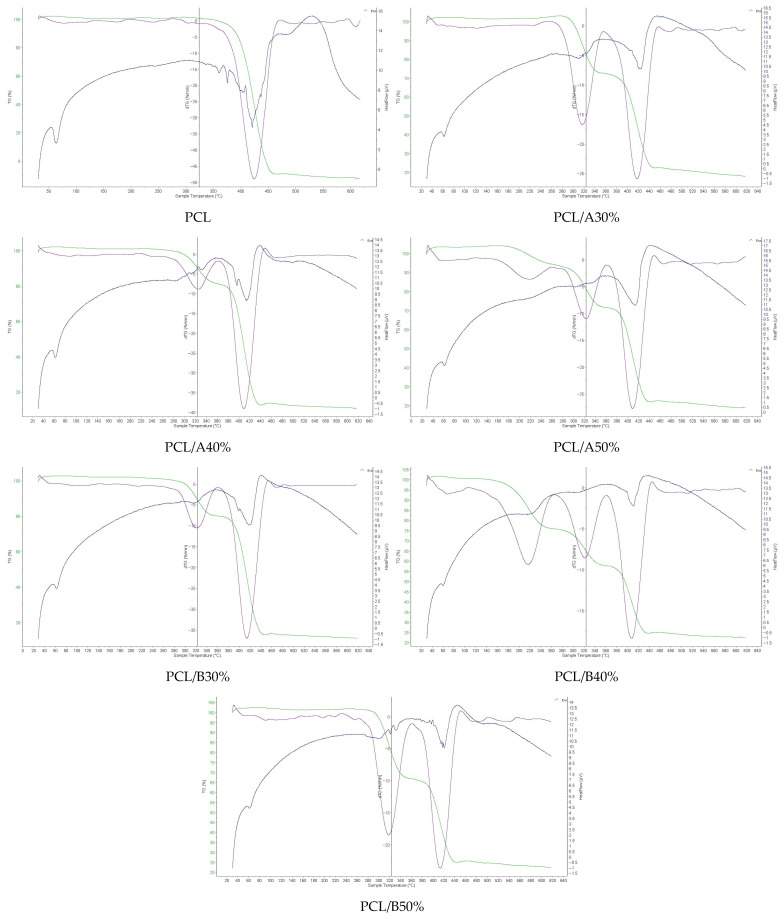
DSC curves of starch–PCL composite films with different starch types and concentrations.

Thermal stability of the films was evaluated by TGA/DTG and DSC (Figure 2). Neat PCL showed a single major decomposition event between 350–460 °C, with maximum weight-loss rate at ~425 °C and a sharp DSC endothermic melting peak near 60 °C, consistent with its semicrystalline structure [48,50].

The incorporation of starch reduced the onset degradation temperature by 10–40 °C, indicating decreased thermal stability. This reduction has been widely reported for starch–polyester composites, where starch acts as a thermally less stable phase [51,52,53]. Multi-stage degradation profiles were observed, reflecting contributions from both starch and PCL. For instance, PCL/B50% exhibited a two-step decomposition with DTG maxima at 318 °C (starch) and 411 °C (PCL), while PCL/A50% displayed a more complex three-step profile (220, 320, and 405 °C), suggesting multiple degradation pathways.

The earlier onset of degradation in A-type starch composites suggests stronger polymer–starch interactions, which alter the decomposition mechanism. Similar findings were reported in starch–PLA composites, where higher substitution degrees led to multi-stage degradation and lower thermal stability [51,54]. B-type starch composites, while less thermally stable overall, exhibited more distinct phase separation between starch and PCL degradation peaks, reflecting weaker polymer interactions.

From an application standpoint, reduced thermal stability may limit high-temperature processing of starch–PCL composites. However, this property is advantageous for biodegradability, as lower decomposition temperatures facilitate microbial and enzymatic breakdown during disposal. Therefore, A-type starch composites may be more suitable for packaging applications requiring mechanical stability at moderate temperatures, whereas B-type composites are better aligned with applications prioritizing rapid thermal and biological degradation.

### 3.7. Water-Resistance Properties

Film thickness, adsorption rate, and WVP are summarized in Table 6. As expected, film thickness increased with starch content for both A- and B-type composites. A-type starch composites exhibited larger thickness increments (208 → 815 μm) compared to B-type (109 → 668 μm), which suggests better polymer–starch adhesion and denser structures. Similar correlations between starch content and film thickness have been reported in PLA–starch and sugar palm starch composites [36,38].

Water adsorption rates increased with starch loading due to the intrinsic hydrophilicity of starch granules. A-type composites showed a consistent rise (11.08 → 17.60 m/min), while B-type films exhibited a non-linear profile, with reduced adsorption at 40% starch (6.09 m/min), possibly due to temporary pore blockage by swollen starch domains. This phenomenon was also observed in starch–PCL and starch–PLA blends, where structural reorganization altered water diffusion pathways [52,53,55,56].

WVP values further highlighted structural differences between starch types. A-type composites exhibited moderate WVP (15–190 g·mm/m^2^·day·kPa), consistent with denser matrices and stronger interfacial adhesion. In contrast, B-type films displayed sharp WVP increases at higher starch loadings (21–215), reflecting their looser microstructure and enhanced porosity, which facilitate vapor transport [57,58].

From an application perspective, A-type starch composites are better suited for moisture-sensitive foods (e.g., bakery items), providing improved water retention and controlled permeability. Conversely, B-type starch films, with higher WVP and porosity, are advantageous for breathable packaging of fresh produce, where water vapor exchange prevents condensation and spoilage.

### 3.8. Antibacterial Activity of Composite Films

**Figure 3:**
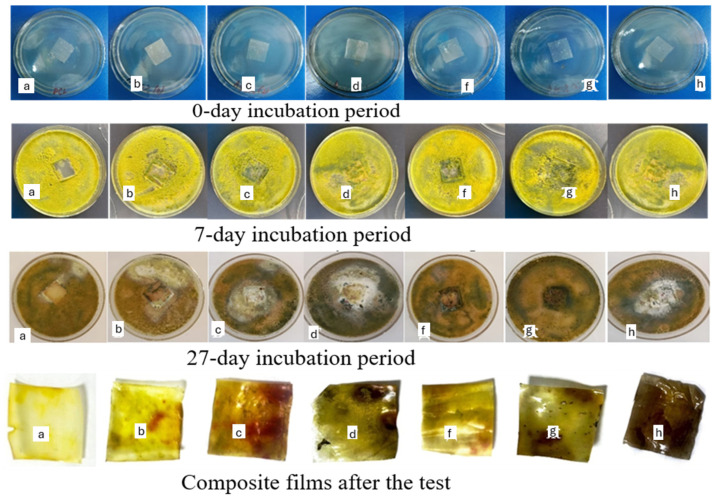
Growth of soil mold fungi on the surface of composite films containing different starch types and concentrations after 27 days of incubation (a—PCL, b—PCL/A30%, c—PCL/A40%, d—PCL/A50%, f—PCL/B30%, g—PCL/B40%, h—PCL/B50%).

Antibacterial performance against E. coli and S. aureus is shown in Table 7 and Figure 3. The inhibition zones increased with starch content for both starch types, confirming starch incorporation improved antibacterial activity. A-type starch composites showed significantly stronger inhibition than B-type films at equivalent loadings (e.g., A50%: 14.6–15.3 mm vs. B50%: 11.2–12.0 mm). This can be explained by higher surface hydrophilicity and better dispersion of A-type starch in the PCL matrix, which facilitate release of acetyl groups or other active compounds capable of disrupting bacterial membranes [18,31]. Similar antibacterial enhancements were reported in acetylated cassava and yam starch films.

Antifungal resistance tests (ISO EN 846) revealed a contrasting trend. Mold colonization was minimal on neat PCL (score 2), but progressively increased with starch loading. At 50% starch, both A- and B-type composites were fully overgrown (score 5), with Trichoderma viride showing the fastest colonization. B-type starch composites were particularly vulnerable, likely due to their smaller granule size and higher nutrient availability, which favor enzymatic degradation and fungal growth. These observations are in agreement with Tian et al. [46], who reported higher fungal susceptibility in starch-rich composites.

Overall, starch incorporation enhanced antibacterial activity but reduced antifungal resistance at high loadings. The best compromise was observed in films with 30–40% starch, particularly A-type composites, which provided effective inhibition against foodborne bacteria while maintaining moderate resistance to mold. Such formulations are suitable for short- to medium-term food packaging applications where bacterial safety is critical, while long-term storage would require additional antifungal strategies (e.g., natural essential oil incorporation).

### 3.9. Biodegradation Properties of Composite Films Under Soil and Composting Conditions

**Figure 4:**
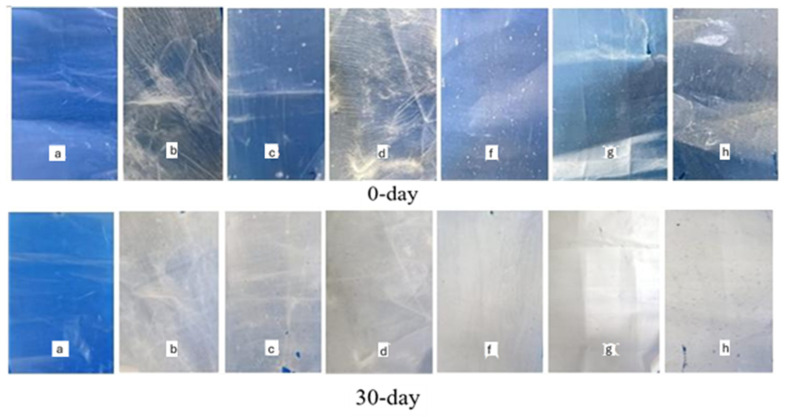
Degradation of composite films in natural soil conditions after 0 and 30 days (a—PCL, b—PCL/A30%, c—PCL/A40%, d—PCL/A50%, f—PCL/B30%, g—PCL/B40%, h—PCL/B50%).

**Figure 5:**
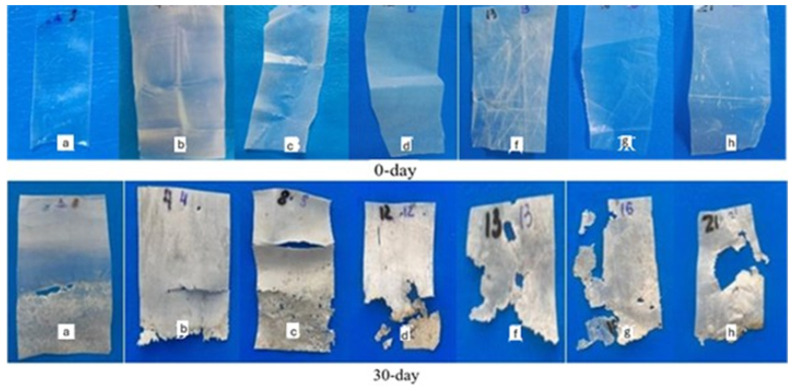
Degradation of composite films in compost conditions after 0 and 10 days. (a—PCL, b—PCL/A30%, c—PCL/A40%, d—PCL/A50%, f—PCL/B30%, g—PCL/B40%, h—PCL/B50%).

The biodegradation behavior of the composite films was evaluated under both natural soil and controlled composting conditions (ISO 17556:2019; ASTM D5338-15). Results are summarized in Table 8 and Figure 4 and Figure 5.

After 30 days of natural soil burial, neat PCL remained largely intact, with only ~1% weight loss, consistent with its known resistance to microbial degradation due to its semicrystalline structure and hydrophobicity [59]. Incorporation of acetylated starch enhanced degradation, with the effect more pronounced in B-type starch composites. For instance, PCL/A50% retained 97.91% of its mass, whereas PCL/B50% retained only 75.37%, indicating higher susceptibility of B-type starch to soil microbes. This behavior can be attributed to the looser crystalline arrangement and higher amylopectin content of B-type starch, which increase water uptake and enzymatic accessibility [57].

These results are in line with previous findings on starch–polyester blends, where starch domains acted as “microbial entry points”, initiating localized hydrolysis and subsequently accelerating polymer fragmentation [40,60].

Composting significantly accelerated degradation due to elevated microbial activity and optimal moisture/temperature. After 10 days, neat PCL still retained >94% of its mass, while starch composites degraded substantially faster (Figure 5). For example, PCL/A40% and PCL/B40% retained only 62.18% and 58.72% of their initial weights, respectively. After 30 days, PCL/B50% showed the most pronounced degradation, retaining just 25.42% of its mass.

Visual observations supported these data: A-type composites became opaque with surface cracks, whereas B-type films exhibited more severe fragmentation and discoloration, suggesting faster starch hydrolysis and subsequent destabilization of the PCL matrix. Such multi-stage degradation (starch domain breakdown followed by polyester fragmentation) is consistent with previously reported mechanisms in starch/PCL and starch/PLA composites [36,41].

Overall, the incorporation of dual-modified starch improved the biodegradability of PCL films, with the effect strongly dependent on starch type and content. B-type starch composites degraded more rapidly, making them suitable for short-term packaging applications requiring rapid composting (e.g., single-use produce packaging). In contrast, A-type starch composites exhibited slower but controlled degradation, balancing durability with environmental safety, which makes them more suitable for medium-term applications such as bakery or snack food packaging [61,62,63].

These findings highlight the potential of starch modification strategies to tune biodegradation rates depending on intended application and waste management conditions. Future work could explore synergistic approaches (e.g., essential oil incorporation, enzymatic pretreatment) to further optimize both antimicrobial and compostability profiles.

### 3.10. Morphological Characterization of Films After Biodegradation

The morphological evolution of neat PCL and starch–PCL composites after 30 days of degradation in soil and compost was examined by SEM (Figure 4 and Figure 5). Prior to degradation, neat PCL films exhibited smooth and homogeneous surfaces with no visible defects, consistent with its semicrystalline structure. After 30 days, only limited surface changes were observed: compost burial induced small cracks and perforations, while natural soil caused shallow fissures. This is in line with the well-documented recalcitrance of PCL under ambient conditions, where microbial attack is restricted due to its hydrophobic backbone [58].

The addition of acetylated A-type wheat starch significantly accelerated biodegradation. After composting, PCL/A30% films exhibited irregular pits (5–15 μm) and interconnected pores, while PCL/A40% showed larger cavities (20–50 μm) localized in starch-rich domains, as confirmed by EDX analysis. These features are typical of starch–polyester blends where starch acts as the preferential microbial target, initiating hydrolysis and opening diffusion channels [41,47]. In natural soil, however, the same composites displayed only superficial etching, highlighting the role of compost conditions (temperature, microbial density, and moisture) in promoting degradation.

B-type starch composites showed even more pronounced structural deterioration. At 30% loading, compost-exposed PCL/B30% films exhibited pits matching the size of the original granules, while at 50% loading (PCL/B50%) the removal of starch domains left crater-like voids and a porous polymer skeleton. This rapid fragmentation can be explained by the smaller granule size, higher swelling capacity, and looser crystalline packing of B-type starch, which enhance water penetration and enzymatic hydrolysis [59,60]. Such microstructural instability increases susceptibility to microbial colonization and accelerates matrix collapse [64,65,66,67].

**Figure 6:**
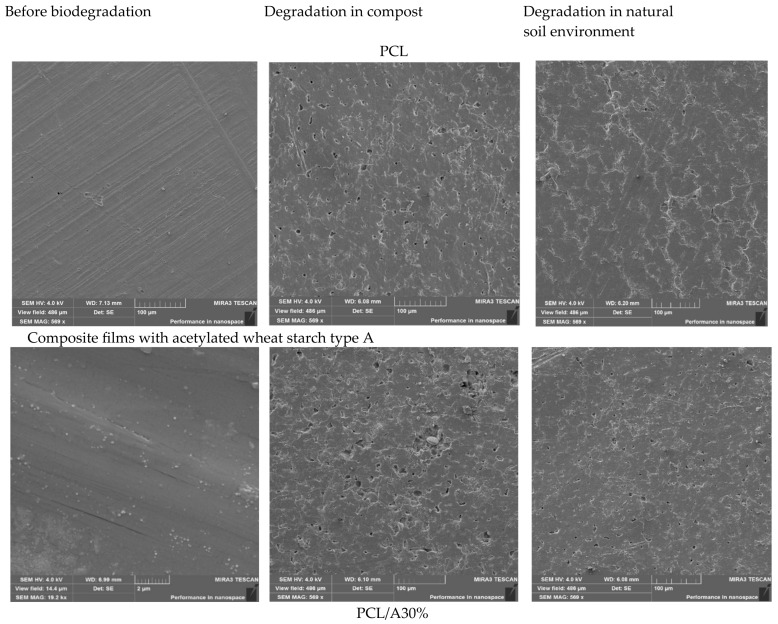

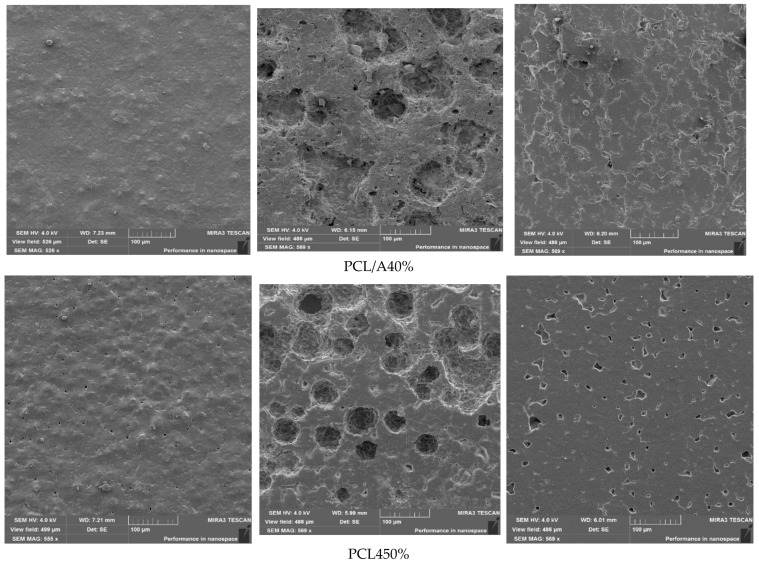
SEM images of PCL and composite films with acetylated wheat starch A before and after degradation in compost and natural soil.

**Figure 7:**
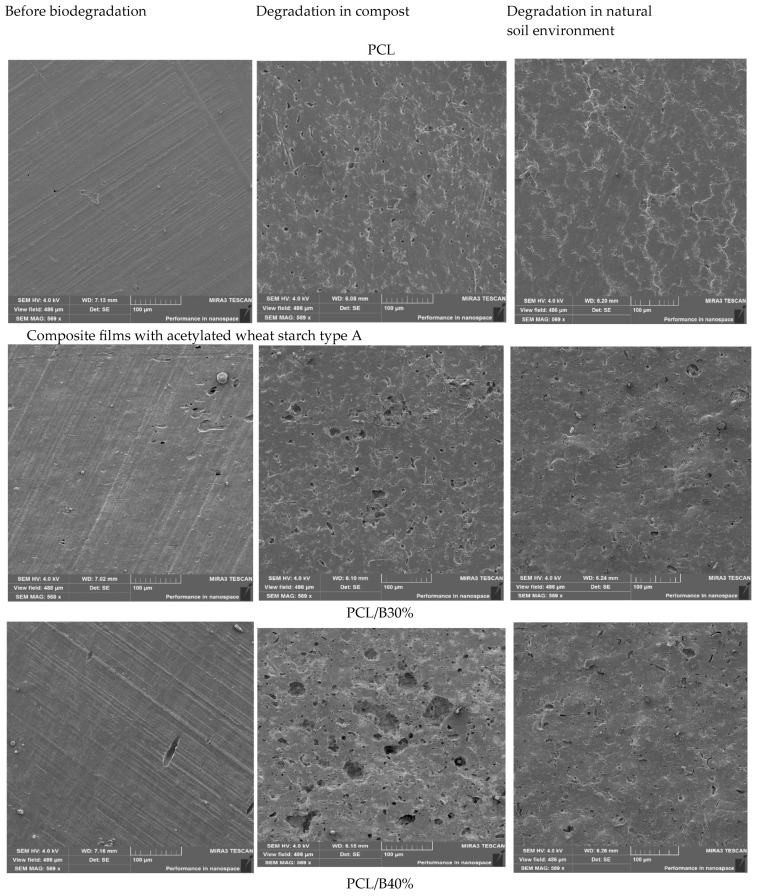

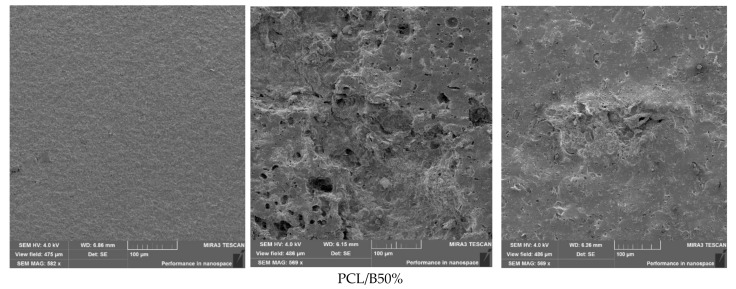
SEM images of PCL and composite films with acetylated wheat starch B before and after degradation in compost and natural soil.

Overall, composting induced far more extensive damage than natural soil burial (Figure 6 and Figure 7), confirming that controlled compost environments provide favorable conditions for biodegradation of starch–polyester systems [36,38]. The SEM findings corroborate the weight-loss data (Section 3.9), confirming that starch domains act as primary microbial entry sites. B-type starch composites degraded faster and more severely than A-type counterparts, underscoring their potential for short-term packaging requiring rapid composting. By contrast, A-type starch composites maintained more structural integrity, making them better suited for medium-term applications such as bakery or snack packaging [68,69].

## 4. Conclusions

In this study, biodegradable composite films were successfully developed by combining polycaprolactone (PCL) with acetylated A- and B-type wheat starches derived from Kazakhstani sources. The results demonstrated that the crystalline type of starch plays a decisive role in determining the structure, properties, and biodegradation behavior of the films.

Acetylated A-type starch formed smoother and more continuous films with higher tensile strength and moderate water vapor permeability, making them suitable for applications requiring mechanical integrity and controlled moisture transfer. In contrast, B-type starch generated more porous structures with higher permeability and faster biodegradation, indicating potential for breathable and rapidly compostable packaging.

Films containing 40–50% acetylated starch achieved an optimal balance between flexibility, barrier function, and biodegradability. The incorporation of starch slightly decreased thermal stability but improved environmental degradability, allowing tailored applications depending on the required shelf life and disposal conditions. Antibacterial tests confirmed that the films exhibited intrinsic inhibitory activity against Escherichia coli and Staphylococcus aureus, supporting their relevance to HACCP-oriented food safety packaging.

Overall, this work demonstrates the feasibility of utilizing regionally available wheat starches to design functional biodegradable films with targeted performance. The findings contribute to sustainable packaging development by offering clear design guidelines for selecting starch types and compositions according to product requirements and environmental goals. Future research will focus on scaling up the process, evaluating long-term stability, and enhancing functionality through the incorporation of natural bioactive agents.

## Figures and Tables

**Table 1:** Physicochemical properties of native and dual-modified (irradiation + acetylation) wheat starches (mean ± SD, n = 3).

Starch Type	Modification	Moisture (%)	Ash (%)	pH	Bulk Density (g/mL)	WAC (g/g)	OAC (g/g)
Wheat A	Native	10.5 ± 0.2 ^b^	0.21 ± 0.01 ^b^	6.72 ± 0.05 ^b^	0.61 ± 0.01 ^a^	0.84 ± 0.03 ^b^	0.72 ± 0.04 ^b^
Wheat A	Dual-modified	9.9 ± 0.1 ^a^	0.18 ± 0.01 ^a^	6.55 ± 0.04 ^a^	0.58 ± 0.02 ^a^	1.22 ± 0.05 ^a^	0.91 ± 0.06 ^a^
Wheat B	Native	11.4 ± 0.3 ^b^	0.24 ± 0.01 ^b^	6.54 ± 0.06 ^b^	0.57 ± 0.01 ^a^	0.89 ± 0.04 ^b^	0.69 ± 0.05 ^b^
Wheat B	Dual-modified	10.2 ± 0.2 ^a^	0.19 ± 0.02 ^a^	6.41 ± 0.05 ^a^	0.55 ± 0.01 ^a^	1.15 ± 0.06 ^a^	0.87 ± 0.04 ^a^

Note: Different superscript letters within the same column indicate statistically significant differences (*p* < 0.05, one-way ANOVA followed by Duncan’s multiple range test). Abbreviations: WAC, water absorption capacity; OAC, oil absorption capacity.

**Table 2:** Functional properties of native and dual-modified (irradiation + acetylation) starches (mean ± SD, n = 3).

Starch Type	Modification	WS (g/100 g)	SP (%)	DS
Wheat A	Dual-modified	3.28 ± 0.13 ^b^	5.88 ± 0.01 ^b^	1.95 ± 0.04 ^a^
Wheat B	Dual-modified	3.28 ± 0.13 ^b^	7.76 ± 0.01 ^a^	1.08 ± 0.03 ^b^
Corn	Dual-modified	8.09 ± 0.11 ^a^	5.93 ± 0.01 ^b^	0.88 ± 0.02 ^c^

Note: Different superscript letters within the same column indicate statistically significant differences (*p* < 0.05, one-way ANOVA followed by Duncan’s test). Abbreviations: WS, water solubility; SP, swelling power; DS, degree of substitution.

**Table 3:** Effect of plasticizer type and concentration on elongation at break of acetylated starch films (mean ± SD, n = 3).

Starch Type	Plasticizer	10%	20%	30%	40%
Wheat A	PVA	31.53 ±6.69 ^d^	28.56 ±5.78 ^c^	23.23 ±6.34 ^b^	15.10 ±5.56 ^a^
Wheat A	Glycerol	41.57 ±4.36 ^a^	38.74 ±3.45 ^a^	31.68 ±5.67 ^c^	22.85 ±4.87 ^b^
Wheat B	PVA	35.66 ±4.79 ^a^	32.42 ±4.48 ^a^	28.35 ±5.22 ^ab^	23.13 ±4.85 ^b^
Wheat B	Glycerol	48.32 ±4.27 ^a^	43.53 ±4.43 ^a^	38.37 ±5.44 ^ab^	34.73 ±5.22 ^b^
Corn	PVA	33.27 ±3.69 ^a^	31.36 ±4.12 ^a^	26.34 ±3.95 ^c^	18.61 ±4.23 ^b^
Corn	Glycerol	44.24 ±3.83 ^a^	41.88 ±4.36 ^a^	35.46 ±4.76 ^b^	27.47 ±4.56 ^c^

Note: Different superscript letters within the same row indicate statistically significant differences (*p* < 0.05, ANOVA + Duncan’s). Abbreviations: PVA, polyvinyl alcohol.

**Table 4:** Selected compositions and tensile strength of PCL/B-type starch composites (mean ± SD, n = 3).

Starch (%)	Glycerol (%)	CaCO_3_ (%)	Measured MPa	Predicted MPa
20	10	5	26.47 ± 0.27	26.48
40	10	5	21.35 ± 0.17	21.56
60	10	5	16.23 ± 0.10	16.34

**Table 5:** Optical transparency of PCL–starch composite films measured at 600 nm (mean ± SD, n = 3).

Sample	Transparency (%)
Neat PCL	91.3 ± 1.2 ^a^
PCL/A30% + 10% glycerol	74.6 ± 2.1 ^b^
PCL/A40% + 10% glycerol	62.7 ± 1.8 ^c^
PCL/A50% + 10% glycerol	48.9 ± 2.5 ^d^
PCL/B30% + 10% glycerol	69.2 ± 1.9 ^b^
PCL/B40% + 10% glycerol	55.8 ± 2.2 ^c^
PCL/B50% + 10% glycerol	41.5 ± 2.7 ^d^

Note: Different superscript letters indicate significant differences (*p* < 0.05, ANOVA + Duncan’s).

**Table 6:** Water-resistance characteristics of starch–PCL composite films (mean ± SD, n = 3).

Sample Composition	Film Thickness (μm)	Adsorption Rate (m/min)	WVP (g·mm/m^2^·day·kPa)
PCL (control)	86 ± 7 ^d^	4.66 ± 0.44 ^c^	11 ± 0.27 ^c^
PCL/A30%	208 ± 27 ^c^	11.08 ± 0.34 ^b^	15 ± 0.54 ^c^
PCL/A40%	422 ± 5 ^b^	16.11 ± 0.43 ^a^	23 ± 0.79 ^c^
PCL/A50%	815 ± 13 ^a^	17.60 ± 0.34 ^a^	190 ± 7.22 ^b^
PCL/B30%	109 ± 32 ^d^	9.68 ± 0.24 ^c^	21 ± 0.61 ^c^
PCL/B40%	268 ± 3 ^c^	6.09 ± 0.40 ^c^	106 ± 4.68 ^b^
PCL/B50%	668 ± 3 ^b^	13.86 ± 0.46 ^b^	215 ± 8.66 ^a^

Note: Values are expressed as mean ± standard deviation (n = 3). Different superscript letters within the same column indicate statistically significant differences according to Duncan’s multiple range test (*p* < 0.05).

**Table 7:** Inhibition zone diameters (mm) of starch–PCL composite films against E. coli and S. aureus (mean ± SD, n = 3).

Sample	*E. coli*	*S. aureus*
PCL	6.0 ± 0.5 ^a^	6.2 ± 0.3 ^a^
PCL/A30%	9.1 ± 0.4 ^b^	10.3 ± 0.5 ^b^
PCL/A40%	11.8 ± 0.3 ^c^	12.5 ± 0.6 ^c^
PCL/A50%	14.6 ± 0.2 ^d^	15.3 ± 0.4 ^d^
PCL/B30%	7.8 ± 0.4 ^ab^	8.5 ± 0.5 ^ab^
PCL/B40%	9.4 ± 0.3 ^b^	10.1 ± 0.4 ^b^
PCL/B50%	11.2 ± 0.3 ^c^	12.0 ± 0.5 ^c^

Note: Different superscript letters indicate significant differences (*p* < 0.05, ANOVA + Duncan’s).

**Table 8:** Retained mass (%) of composite films with different starch types and concentrations during soil burial and composting (mean ± SD, n = 3).

Sample	Natural Soil (%)				Compost Soil (%)			
	Day 0	Day 10	Day 20	Day 30	Day 0	Day 5	Day 10	Day 30
PCL	100 ± 0.00 ^a^	99.35 ± 0.01 ^a^	99.19 ± 0.02 ^a^	98.95 ± 0.02 ^a^	100 ± 0.00 ^a^	96.33 ± 0.01 ^a^	94.69 ± 0.02 ^a^	92.15 ± 0.03 ^a^
PCL/A30%	100 ± 0.00 ^a^	99.40 ± 0.02 ^a^	99.22 ± 0.08 ^a^	99.02 ± 0.03 ^a^	100 ± 0.00 ^a^	75.24 ± 0.11 ^b^	64.08 ± 0.13 ^b^	42.67 ± 0.18 ^b^
PCL/A40%	100 ± 0.00 ^a^	99.18 ± 0.04 ^a^	98.82 ± 0.08 ^a^	98.40 ± 0.05 ^a^	100 ± 0.00 ^a^	82.56 ± 0.02 ^b^	62.18 ± 0.10 ^b^	40.12 ± 0.15 ^b^
PCL/A50%	100 ± 0.00 ^a^	98.11 ± 0.08 ^a^	97.99 ± 0.07 ^a^	97.91 ± 0.06 ^a^	100 ± 0.00 ^a^	76.88 ± 0.07 ^b^	56.25 ± 0.06 ^b^	33.54 ± 0.14 ^b^
PCL/B30%	100 ± 0.00 ^a^	89.82 ± 0.02 ^b^	89.65 ± 0.05 ^b^	89.42 ± 0.01 ^b^	100 ± 0.00 ^a^	84.31 ± 0.06 ^b^	73.35 ± 0.07 ^b^	49.12 ± 0.17 ^b^
PCL/B40%	100 ± 0.00 ^a^	82.64 ± 0.04 ^b^	82.40 ± 0.01 ^b^	82.14 ± 0.03 ^b^	100 ± 0.00 ^a^	75.83 ± 0.09 ^b^	58.72 ± 0.12 ^b^	29.86 ± 0.16 ^c^
PCL/B50%	100 ± 0.00 ^a^	75.65 ± 0.08 ^c^	75.49 ± 0.08 ^c^	75.37 ± 0.03 ^c^	100 ± 0.00 ^a^	71.67 ± 0.06 ^c^	55.29 ± 0.09 ^c^	25.42 ± 0.13 ^c^

Note: Table reports retained mass (%); lower values indicate faster biodegradation. Different superscript letters within the same column indicate statistically significant differences (*p* < 0.05, one-way ANOVA + Duncan’s multiple range test).

## Data Availability

The original contributions presented in this study are included in the article. Further inquiries can be directed to the corresponding authors.

## Abbreviations

The following abbreviations are used in this manuscript:

PCL	Polycaprolactone
WVP	Water Vapor Permeability
SEM	Scanning Electron Microscopy
DSC	Differential Scanning Calorimetry
TGA	Thermogravimetric Analysis
FTIR	Fourier Transform Infrared Spectroscopy
CFU	Colony Forming Units
RH	Relative Humidity
ISO	International Organization for Standardization
A-type starch	Acetylated wheat starch (type A)
B-type starch	Acetylated wheat starch (type B)
ASTM	American Society for Testing and Materials
SD	Standard Deviation
ANOVA	Analysis of Variance
HACCP	Hazard Analysis Critical Control Point
WAC	Water absorption capacity
OAC	Oil absorption capacity
WS	Water Solubility
SP	Swelling Power
DS	Degree of Substitution
UV–Vis	Ultraviolet–Visible Spectroscopy
EDX	Energy Dispersive X-ray Spectroscopy
